# Mpox among Public Festival Attendees, Chicago, Illinois, USA, July–August 2022

**DOI:** 10.3201/eid2905.221797

**Published:** 2023-05

**Authors:** Emily A.G. Faherty, Richard A. Teran, Stephanie R. Black, Vaishali Chundi, Shamika Smith, Brandon Bernhardt, Emma Weber, Bridget Brassil, Peter Ruestow, Janna L. Kerins

**Affiliations:** Centers for Disease Control and Prevention, Atlanta, Georgia, USA (E.A.G. Faherty);; Chicago Department of Public Health, Chicago, Illinois, USA (E.A.G. Faherty, R.A. Teran, S.R. Black, V. Chundi, S. Smith, B. Bernhardt, E. Weber, B. Brassil, P. Ruestow, J.L. Kerins)

## Abstract

We investigated an mpox outbreak after a 2022 LGBTQ event in Chicago, Illinois, USA. Among case-patients, 38% had received 1 dose of mpox vaccine, none 2 doses; most reported sexual activity during the probable exposure period. Among other preventive measures, persons at risk should complete mpox vaccination 14 days before an event.

Monkeypox virus transmission may be associated with large events where at-risk or potentially infected persons engage in sexual activity at or outside the event (*1*). The Chicago Department of Public Health (CDPH) investigated an mpox outbreak associated with Market Days (MD), an annual LGBTQ outdoor festival held in Chicago, Illinois, USA, August 6–7, 2022, attended by ≈100,000 persons from across the country. We describe persons with mpox who attended the festival.

CDPH interviewed mpox case-patients (*2*) to ascertain MD attendance and conducted a supplemental survey about exposures, sexual behavior, and preventive measures. Event-attending case-patients were defined as persons with positive mpox or orthopoxvirus test results who attended MD on August 6–7 and had symptoms within 21 days before or after attendance (July 16–August 28). Illinois surveillance systems verified laboratory and JYNNEOS (https://www.jynneos.com) vaccination data. CDPH also solicited cases from other jurisdictions in a national call for cases related to the event. Event-associated case-patients were categorized by vaccination status at the time of MD. This investigation was reviewed by the Centers for Disease Control and Prevention and conducted in accordance with its policies and applicable federal law (45 C.F.R. part 46.102(l) (*2*), 21 C.F.R. part 56; 42 U.S.C. §241(d); 5 U.S.C. §552a; 44 U.S.C. §3501 et seq).

We identified 40 event-associated case-patients from Chicago and other jurisdictions who attended MD; symptom onset occurred before MD for 7 and during or after for 33 (Figure). Case-patients with symptom onset before MD had a median of 1 (interquartile range [IQR] 1–2) sex partner during MD, and case-patients with symptom onset after MD had a median of 5 (IQR 2–5) sex partners during MD (Appendix). Many case-patients were non-Hispanic/Latino White (45%), most were male at birth (98%), and most reported having sex with men (70%) (Table). Two case-patients, 1 living with HIV, were hospitalized for mpox. 

**Figure:**
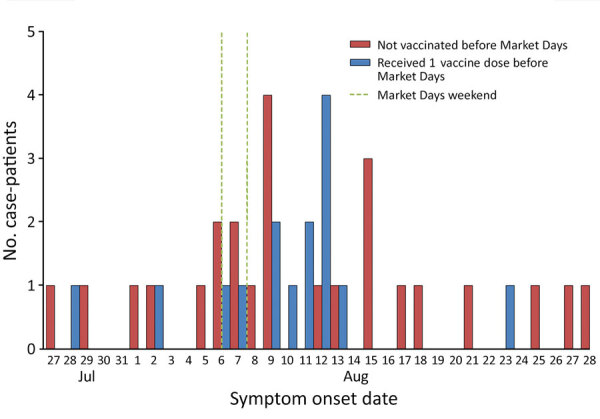
Epidemic curve for mpox case-patients who attended Market Days event, by symptom onset date, Chicago, Illinois, USA, July–August 2022. Vertical dashed lines indicate dates of Market Days event. Vaccine was JYNNEOS (https://www.jynneos.com).

**Table:** Characteristics of mpox case-patients attending MD event, by vaccination status before the event, Chicago, Illinois, USA, July–August 2022*

Characteristics	All cases, n = 40	Vaccinated before MD, n = 15	Not vaccinated before MD, n = 25
Demographics†			
Chicago resident	36 (90)	14 (93.3)	22 (88)
Male sex at birth	39 (97.5)	15 (100)	24 (96)
Median age in years, IQR	31 (27.3–35.8)	32 (29–35.5)	30 (25.5–35.5)
Reported male-to-male sexual contact	28 (70)	11 (73.3)	17 (68)
Race/ethnicity			
Non-Hispanic or Latino White	18 (45)	8 (53.3)	10 (40)
Non-Hispanic or Latino Black	9 (22.5)	2 (13.3)	7 (28)
Hispanic or Latino	13 (32.5)	5 (33.3)	8 (32)
Sexual exposures			
Reported having sex during probable exposure period	32 (80)	12 (80)	20 (80)
Reported having sex during MD	14 (35)	6 (40)	8 (32)
Sex with a main partner at MD	1 (2.5)	-	1 (4)
Sex with a casual partner at MD	3 (7.5)	1 (6.7)	2 (8)
Sex with an anonymous partner at MD	7 (17.5)	4 (26.7)	3 (12)
Reported nonsexual skin-to-skin contact or kissing during probable exposure period	4 (10)	2 (13.3)	2 (8)
Reported sexual activity outside probable exposure period	1 (3)	ND	1 (4)
Missing information about sexual exposures	1 (3)	ND	1 (4)
Median no. sex partners at MD (IQR)	3.5 (1.3–5)	4 (2.3–5)	3 (1–5.3)
Met sex partners at MD	9 (23)	4 (26.6)	5 (20)
Met at another location	2 (5)	2 (13.3)	ND
Met by using an online app	2 (5)	2 (13.3)	ND
Nonvaccine prevention measures‡	28 (70)	12 (80)	16 (64)
Abstained from sex	9 (23)	2 (13.3)	7 (28)
Reduced number of sex partners	5 (13)	2 (13.3)	3 (12)
Avoided skin-to-skin contact	8 (20)	4 (26.7)	4 (16)
Wore more clothing	3 (8)	2 (13.3)	1 (4)
Other measures§	8 (20)	3 (20)	5 (20)
*Values are no. (%) except as indicated. Tests of significant differences by vaccination status were conducted by using Fisher exact tests and t-tests for continuous variables; no values were p>0.05. IQR, interquartile range; MD, Market Days; ND, no data. †Percentages may not add up to 100% because of rounding. ‡Case-patients may have reported adopting >1 prevention measure. §Other measures included isolating after the event and wearing protective clothing or a mask.			

Fifteen (38%) case-patients had received 1 dose of JYNNEOS vaccine before MD; none received 2 doses or were due for their second dose by MD (within 28 days of first dose). Among 1-dose recipients, 8 (53%) were vaccinated <14 days before symptom onset. Among all 1-dose recipients vaccinated before symptom onset, median time from vaccination to symptom onset was 13 (IQR 9–29) days. Fewer non-Hispanic/Latino Black case-patients were vaccinated before MD (2/15, 13%) than non-Hispanic/Latino White (8/15, 53%) or Hispanic/Latino (5/15, 33%) case-patients.

Most (32/40, 80%) case-patients reported having had sexual activity during the probable exposure period (21 days before symptom onset). Case-patient reports included sexual activity during MD (August 6–7) at any location, meeting a partner at MD, and sexual activity at the event. Only 14 (35%) case-patients reported sexual activity during MD at any location, including 3 with symptom onset before those dates. Nine (23%) persons engaged in sex with >1 partner during MD, 9 (23%) met a sex partner at MD, and 4 (10%) case-patients reported sexual activity at MD. Case-patients reporting sexual activity during MD had a median of 3.5 (IQR 1.3–5.0) partners; more had anonymous sex partners (7/14, 50%) than casual or main partners (4/14, 29%). More case-patients who received 1 vaccine dose before MD had sex with an anonymous partner during MD (4/15, 27%) than did unvaccinated case-patients (3/25, 12%).

Among all event-associated case-patients, 28 (70%) adopted nonvaccine prevention measures during MD; 9 (23%) abstained from sex, and 8 (20%) avoided skin-to-skin contact. More case-patients unvaccinated before MD abstained from sex during MD (7/25, 28%) than did 1-dose recipients (2/15, 13%). Seven (78%) of 9 case-patients who abstained from sex during MD reported having sex during the probable exposure period. Among case-patients reporting sexual activity during MD, 2 (2/14, 14%) reduced their number of sex partners and 4 (4/14, 29%) adopted other measures (e.g., isolating after MD).

Although MD might have presented more opportunities for sex partnerships, <50% of case-patients reported sex related to the event. Among case-patients who had sex during MD, most had anonymous sex partners, potentially increasing transmission (*3*). Most case-patients also reported adopting >1 prevention measure during MD but may not have maintained measures throughout the probable exposure period. For example, among 9 persons who abstained from sex at MD, 7 (78%) reported sexual activity with >1 partner outside the event.

More than one third of case-patients reported 1 vaccine dose before MD. Symptoms developed within 14 days after the first dose for most (8, 53%) who were partially vaccinated before MD. Although equal proportions of vaccinated and unvaccinated persons reported sex during MD, 1-dose recipients reported taking fewer precautions, such as abstaining from sex or avoiding anonymous sex.

Among study limitations, we may have undercounted event-associated case-patients. Out-of-state case-patients may not have reported or been asked about event attendance. Also, without a comparison group, we could not compare preventive measures by case-patient status. Information about prevention measures and sexual behavior were self-reported and subject to social desirability bias.

Vaccination uptake before large gatherings may affect behavior and perceived infection risk. Risk messaging should emphasize completing vaccination 14 days before an event and taking other measures to prevent mpox.

Appendix Characteristics of mpox case-patients attending Market Days, by symptom onset timing relative to the event, Chicago, IL, July–August 2022.
