# Supplementary material for: Mpox among Public Festival Attendees, Chicago, Illinois, USA, July–August 2022
Source: Emerg Infect Dis. 2023 May;29(5):1059–61. doi: 10.3201/eid2905.221797 (PMC10124669; doi:10.3201/eid2905.221797)
Supplement: Appendix — Characteristics of mpox case-patients attending Market Days, by symptom onset timing relative to the event, Chicago, IL, July–August 2022. [file 22-1797-Techapp-s1.pdf]

Article DOI: https://doi.org/10.3201/eid2905.221797

EID cannot ensure accessibility for supplementary materials supplied by authors. Readers who have difficulty accessing supplementary content should contact the authors for assistance.

Mpox among Public Festival Attendees, Chicago, Illinois, USA, July–August 2022

Appendix

Appendix Table. Characteristics of case-patients attending Market Days (MD) by symptom onset timing relative to the event — Chicago, IL, July–August 2022

Characteristics*	All cases n=40	Onset before MD n=7	Onset during MD n=6	Onset after MD n=27
Demographics n (%)				
Chicago residents	36 (90%)	7 (100%)	5 (83.3%)	24 (88.9%)
Male sex at birth	39 (97.5%)	7 (100%)	5 (83%)	27 (100%)
Median age in years (IQR)	31 (27.3-35.8)	37 (28.5-43)	34 (30-34)	30 (26.3-34)
Reported male-to-male sexual contact	28 (70%)	5 (71.4%)	2 (33.3%)	21 (77.8%)
Race/ethnicity†				
Non-Hispanic or Latino White	18 (45%)	3 (42.9%)	3 (50%)	12 (44.4%)
Non-Hispanic or Latino Black	9 (22.5%)	2 (28.6%)	-	7 (25.9%)
Hispanic or Latino	13 (32.5%)	2 (28.6%)	3 (50%)	8 (29.6%)
Sexual Exposures n (%)				
Reported having sex in probable exposure period	32 (80%)	5 (71.4%)	2 (33.3%)	25 (92.6%)
Reported having sex during MD	14 (35%)	3 (42.9%)	-	11 (40.7%)
Sex with a main partner at MD	1 (2.5%)	1 (14.3%)	-	-
Sex with a casual partner at MD	3 (7.5%)	1 (14.3%)	-	2 (7.4%)
Sex with an anonymous partner at MD	7 (17.5%)	1 (14.3%)	-	6 (22.2%)
Reported non-sexual skin to skin contact or kissing in probable exposure period	4 (10%)	-	2 (33.3%)	2 (7.4%)
Reported sexual activity outside probable exposure period	1 (2.5%)	1 (14.3%)	-	-
Missing information on sexual exposures	1 (2.5%)	-	1 (16.7%)	-
Median # of sex partners at MD (IQR)	3.5 (1.3-5)	1 (1-1.5)	-	5 (2.5-5.5)
Met sex partners at MD	9 (23%)	2 (28.6%)	-	7 (26%)
Met at another location	2 (5%)	-	-	2 (7.4%)
Met using an app, online	2 (5%)	-	-	2 (7.4%)
Non-Vaccine Prevention Measures Adopted n (%)‡	28 (70%)	4 (57.1%)	4 (66.7%)	20 (74.1%)
Abstained from sex	9 (22.5%)	1 (14.3%)	1 (16.7%)	7 (25.9%)
Reduced number of sex partners	5 (12.5%)	1 (14.3%)	-	4 (14.8%)
Avoided skin-to-skin contact	8 (20%)	1 (14.3%)	1 (16.7%)	6 (22.2%)
Wore more clothing	3 (7.5%)	1 (14.3%)	1 (16.7%)	1 (3.7%)
Other measures§	8 (20%)	1 (14.3%)	2 (33.3%)	5 (18.5%)

*Units are no. (%) unless otherwise indicated. Tests of significant differences were conducted by using Fisher exact tests and t-tests for continuous variables; no values were p>0.05. IQR, interquartile range; MD, Market Days.
†Percentages may not sum to 100% due to rounding.
‡Case-patients may have reported adopting more than one prevention measure.
§Other measures included isolating after the event and wearing protective clothing or a mask.
